# A New Index of Coordinated Posterior and Anterior Evoked EEG to Detect Recall Under Sedation – A Pilot Study

**DOI:** 10.1038/s41598-019-54270-3

**Published:** 2019-11-28

**Authors:** Dana Baron Shahaf, Gregory M. T. Hare, Andrew J. Baker, Violina Chenosia, Leonid Priven, Nikhil Mistry, Goded Shahaf

**Affiliations:** 1 Department of Anesthesia, Rambam Health Care Campus, Haifa, Israel; 2 Department of Anesthesia, St. Michael’s Hospital, University of Toronto, 30 Bond Street, Toronto, Ontario, M5B 1W8 Canada; 3 St. Michael’s Hospital Center of Excellence for Patient Blood Management, Toronto, Canada; 4 Department of Physiology, University of Toronto, Toronto, Ontario, M5S 1A8 Canada; 5 Keenan Research Centre for Biomedical Research, in the Li Ka Shing Knowledge Institute, 209 Victoria Street, Toronto, Ontario, M5B 1T8 Canada; 6 NeuroIndex LTD. Beit Tavor, Yokneam, Israel

**Keywords:** Translational research, Scientific data

## Abstract

EEG-based technologies may be limited in identifying recall under sedation (RUS). We developed a novel index, posteriorization/anteriorization (P/A) index, based on auditory evoked EEG signal and assessed whether it could differentiate between patients with or without RUS. Methods: EEG and BIS were sampled from 3 groups: 1. Patients undergoing sedation (n = 26); 2. Awake volunteers (n = 13, positive control for recall) 3. Patients undergoing general anesthesia (GA, n = 12, negative control for recall). In recovery, recall was assessed using the BRICE questionnaire. Of the 26 sedated patients, 12 experienced recall. Both The P/A index and BIS differentiated between patients with recall and no recall. However, BIS differentiation may have been sensitive to the main drug used for sedation (midazolam vs. propofol) and the P/A index did not show similar drug-based sensitivity. Furthermore, only BIS results were correlated with EMG. Conclusion: This pilot study provided support for the association between P/A index and recall after sedation. Further research is needed in integrating the index into clinical use: (1) it should be derived by an easy-to-use EEG system with a better signal-to-noise ratio; (2) its applicability to other drugs must be shown.

## Introduction

Recall of awareness under anesthesia (AUA) is a serious adverse event with acute and long-lasting impact on patients^1–4^. Various EEG-based technologies have been developed in order to identify this condition during anesthesia^5^. However, over recent years, significant evidence casts doubt regarding the ability of such monitors to identify patients at risk for AUA with high precision, in part due to lack of sensitivity to various hypnotic agents^6^ or the confounding influence of muscle activity, on the EEG signal^7–10^. In addition, the very low prevalence of awareness under general anesthesia (GA)^11–15^ makes it difficult to validate the effectiveness of these monitors.

Alternatively, sedation often involves greater prevalence of recall as defined by the nature of the targeted clinical condition^13,16,17^. The impact of recall under sedation (RUS) is usually not distressing as patients are expecting some degree of awareness and are not paralyzed, hence, they can signal when becoming aware and/or uncomfortable^13,18–22^. Assessing RUS provides us with a study population with an expectedly higher incidence of recall, without impeding the impact of neuromuscular activity on the electroencephalogram (EEG) signal. With the growing use of sedation, by Anesthesiologists and Non-Anesthesiologists, there is a growing need for accurate measurements of anesthesia depth and awareness to enable safety while avoiding the risks associated with inappropriately deep anesthesia (18–20).

We have previously assessed the relationship between anterior and posterior auditory evoked potentials (EP) as a measure of integrated brain function identifying that anterior brain activity is associated with attention processes, while the posterior brain activity is more related to perception processes^23,24^. Published data suggests that lighter levels of anesthesia lead to fragmentation and reduction of attention-related waves and deeper anesthesia results also in the reduction of perception-related waves^25–28^. Other studies have demonstrated that alpha activity anteriorizes under anesthesia^29–31^. Therefore, the phenomenon of alpha anteriorization under anesthesia, might stem initially from anterior fragmentation of attention-related activity and then progress from a posterior decrease of perception-related activity, at deeper levels of anesthesia or sedation. Hence, this study focuses on alpha anteriorization, which may relate to attention and perception processes, as a measure of depth of anesthesia.

To measure attention and perception processes which are displayed in evoked potentials (EP) activity, we used a simple auditory oddball stimulus, which do not require patient’s response, in order to evoke and evaluate the electrophysiological activity. In this proof of concept study, we assessed EP response and generated a novel P/A index based on computerized analysis of the relation between the EP posterior alpha activity to the EP anterior alpha activity. We hypothesized that the new evoked EEG index would differentiate between patients with or without recall under sedation^32,33^.

Thus, our main study question was whether the P/A index would tend to be different between sedated patients who have recall (as identified by Brice questionnaire^34^– see below) and sedated patients who do not have recall. However, sedation is performed with various drugs and it is known different drugs might have different effect on EEG^35^, on the one hand and different likelihood of recall, on the other hand^16,17,36^. Therefore, despite the pilot nature of this study and its limited sample size and characteristics, we sought ways to evaluate, in a very preliminary manner, whether a possible association between the P/A index and recall might be related to drug type.

Furthermore, in order to enhance the Brice-questionnaire based evaluation of recall (35), we also added to the study two control groups – one (awake participants), in which we expected full recall (as indeed was the case) and one (patients undergoing general anesthesia), in which we expected complete lack of recall (as indeed was the case). We analyzed also the P/A index in these two groups as another objective evaluation of an index, which is expected to be associated with recall.

Yet, when one introduces a novel index, even in a very preliminary manner, it seems advisable to evaluate, even in a preliminary manner, to what extent this index is indeed novel in comparison to accepted indices in the field. Thus, we compared the novel P/A index with the prevailing BIS, which was measured simultaneously in all samples. Thus, BIS was also evaluated in terms of (1) differentiation of recall vs. no-recall in sedations, (2) preliminary sensitivity to drug type in this regard and (3) differentiation also with regard of the two control groups specified above.

Finally, as in sedation, frontal EMG can certainly impact the electrophysiological markers, and due to the above-mentioned possibility of such an impact, we further analyzed the impact of EMG on both the P/A index and BIS. This impact analysis and its relation with possible similarities and differences between the indices, with regard to the three study goals mentioned above, was our last study goal. Since the comparison of the P/A index and of BIS with the EMG activity was not formally specified a-priori as one of the study outcomes, it should only be viewed as a post-hoc analysis.

## Methods

### Participants

Following Rambam Health Care Campus institutional ethics approval (152–16) in accordance with the relevant guidelines and regulations, and NIH trial registration (NCT02938325), this prospective study was performed at Rambam campus, Haifa, Israel between January 2017 to June 2018. Patients were recruited following obtaining an informed consent from all participants and/or their legal guardians, if they were: 1) Target group: patients undergoing sedation for various procedures (cardiac electrical studies, liver chemoembolization, biliary duct drainage); 2) First control group**:** awake participants (positive control for recall); or 3) Second control group: patients undergoing abdominal surgery under general anesthesia (GA; negative control for recall). Signed informed consent was obtained from all participants. All recruited patients underwent intra procedural EEG recording and interviewed at the end of the surgery/ procedure. Exclusion recruitment criteria included patient age (<18 years), pregnancy, and patients with ASA IV-V criteria. The samples from the first 10 recruited patients in the study in the GA group were utilized for refining the experimental set-up and data collection, i.e. headset positioning together with the other monitors. These samples were not included in the analysis.

### Study flow

**Figure 1:**
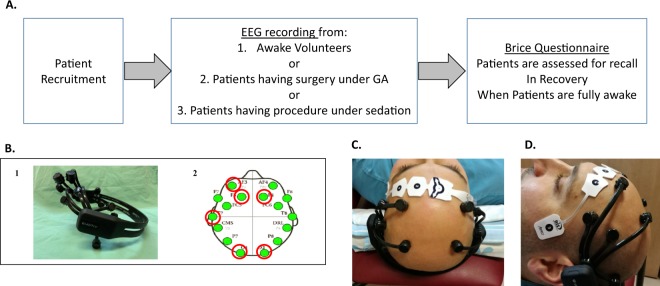
Study Design: Study flow, Emotiv headset and BIS positioning. (**A**) Study flow. B1. Emotiv Epoc Headset. B2. EEG analysis was done for the marked electrodes; Frontal (F3, F4), Posterior (O1, O2). EMG/EOG was analyzed from AF3. All were referenced to left T7. (**B**) A view from above of placing Emotiv headset and BIS on a volunteer’s head. (**C**) A side view of Emotiv headset and BIS on volunteer’s head.

After patients’ recruitment, EEG and BIS were recorded, in parallel, during the surgical procedure (which was done under sedation or general anesthesia), from its beginning until its end. For patients under GA, the EEG helmet and the BIS were placed after patient’s intubation. For patients under sedation, the EEG helmet and BIS placement were done while the patient was connected to all other standard ASA monitoring. Awake volunteers served as a positive control group for recall. Their EEG and BIS were recorded for approximately 10 minutes in supine position, while their eyes were closed, without having any procedure. At the end of the surgery/ procedure, when patients were fully awake at post anesthesia care unit (PACU), before discharge, they were assessed for having recall, using the standard Brice questionnaire^34,37^. The EEG analysis were compared to the results from the BRICE questionnaire (which are summarized to YES/NO recall) (Fig. 1, Appendix 1).

### EEG system

EEG was sampled by Emotiv Epoc 128 Hz system (using saline based electrodes)^38,39^ (Fig. 1) parallel to BIS recordings^40^. We chose Emotiv Epoc since it is a low-cost system, which could be placed quickly on the head even without prior experience in EEG sampling. The system uses saline embedded electrodes without the need for head shaving or gel uses. We used towels to support the head to obtain signal in supine position (Fig. 1C,D). Informed consent for publishing the Volunteer’s picture in an online open-access publication was obtained. Data was sampled from channels F3, F4, O1, O2 and referenced to T7 for signal analysis (Fig. 1B2).

### Assessment of Recall using the Brice questionnaire

As stated previously, When patients were fully awake, before discharge from PACU, they were evaluated for recall using the Brice questionnaire^34,37^ (Appendix 1). The questionnaire included 3 main questions: 1. What is the last thing you remember before going to sleep? 2. What is the first thing you remember after waking up? 3. Do you remember anything between going to sleep and waking up? If patients answered “YES” for the third question, they were further questioned which type of recall they have (i.e. hearing events of the surgery, feeling anxiety or stress, feeling pain, feeling the procedure without pain) and were considered as having recall.

### Sedation and EEG measurements

After signing an informed consent, the participants were sampled with both EEG and BIS in supine position parallel to standard American Society of Anesthesia (ASA) monitoring as part of their management. The study was observational. The anesthesiologists were not instructed to use a specific anesthetic protocol. They also did not view the BIS or raw EEG data in real-time and were not affected by them in their treatment selection. BIS values and were registered manually every 5 minutes using a standard fill-in form, which was prepared in advance. During the sampling period the patients were exposed to auditory stimulation of pure tones of 1000 Hz and of 2000 Hz at 60 DB delivered via earphones. 80% of the stimuli were of the 1000 Hz tone and 20% of the stimuli were of the 2000 Hz tone (oddball protocol – which is often used in anesthesia in one format or the other – e.g. (28)). The order of stimuli was arbitrary, presented every 2–3 seconds, with random delay in that range. [We cannot rule out that auditory stimulation may increase the likelihood of awareness. However, the main question concerned the association between the electrophysiological indices and the occurrence of recall of awareness].

When patients were fully awake, they were evaluated by Brice questionnaire for recall. Patients who reported any recall (voices, touch, pain etc.) were included in the recall group. Patients who did not report any recall were included in the no-recall group. Using the questionnaire, we also evaluated dreaming during the procedure – however, none of the patients in the recall group did not report dreaming.

The sedations were provided without any protocol restriction. The main sedation drug was either Midazolam or Propofol, but often both drugs were used in various doses. In addition, patients received Fentanyl or Remifentanyl, according to the choice of the anesthesia provider (Table 1). About half of the procedures were performed in the Angiography suite and about half of the procedures were performed in the Cardiac suite. In such a small pilot study, there are pros and cons for sampling patients, which undergo two types of procedures. It is obvious that with such a small sample size homogeneity would be of value. However, due to the stated differences in the impact of various anesthetics on the electrophysiological activity, it seemed of value to include already in the initial exploration some variability, which we hoped would enable the sampling of the effects of at-least two major anesthetic drugs (midazolam and propofol). Especially, since we did not want to interfere with the protocols employed. Therefore, we sampled at these two different settings of angiography and cardiac suits. However, in the end there were no major differences between the different settings in terms of the major drug used (see Table 1).

**Table 1:** Baseline and operative characteristics.

	Awake (recall; N = 13)	Midazolam (recall; N = 12)	Midazolam (no recall; N = 6)	Propofol (no recall; N = 8)	General Anesthesia (no recall; N = 12)
**Baseline Characteristics**					
Male Sex, No. (%)	10 (77)	9 (75)	6 (100)	7 (88)	9 (75)
Age - years, median (IQR)	45 (33, 51)	63 (57, 67)	59 (56, 69)	64 (60, 65)	69 (63, 83)
Weight - kg, median (IQR)		77 (72, 82)	77 (66, 82)	71 (70, 75)	78 (68, 79)
Height - cm, median (IQR)		172 (164, 177)	174 (172, 175)	171 (168, 175)	168 (159, 169)
**Operative Characteristics**					
Angio: Cardiac procedures (numbers)		6:6	3:3	5:3	
Duration of recording - minutes, median (IQR)	13 (10.5, 14.8)	77.5 (53.8, 113.8)	57.5 (42.5, 72.5)	72.5 (53.8, 113.8)	86.16 (32.6, 139)
Administered midazolam, No. (%)	0 (0)	10 (83)	6 (100)	8 (100)	0 (0)
Dose - mg, median (IQR)		2.25 (2, 3)	3 (3, 4.5)	1 (1, 2)	0 (0)
Administered fentanyl, No. (%)	0 (0)	10 (83)	5 (83)	8 (100)	12 (100)
Dose - mgc, median (IQR)		100 (81.25, 115)	100 (50, 100)	150 (137.5, 150)	1.5 mcg/kg (<1 mcg/kg)
Administered remifentanyl, No. (%)	0 (0)	3 (25)	1 (17)	0 (0)	0 (0)
Dose - mg, median (IQR)		0.23 (0.23, 0.24)	0.26		
Administered propofol, No. (%)	0 (0)	7 (58)^a^	3 (50)	8 (100)	12 (100)
Dose - mg, median (IQR)		30 (15, 45)	40 (30, 50)	280 (235, 285)	1.5 mg/kg (<1 mg/kg)
Patients with recall, No. (%)	14 (100)	12 (100)	0 (0)	0 (0)	0 (0)
Sample duration (Seconds + SD)	765 ± 134	3423 ± 1434	4158 ± 2077	4564 ± 2323	5289 ± 1766
Inter-A/P index interval (sec)^b^	103 ± 96	165 ± 167	86 ± 33	72 ± 42	297 ± 314

IQR, interquartile range; ^a^One patient received 100 mg Ketamine; ^b^The median interval in seconds between non-noisy segments for which a valid PI value was generated (lower values indicate better signal quality).

IQR, interquartile range; ^a^One patient received 100 mg Ketamine; ^b^The median interval in seconds between non-noisy segments for which a valid PI value was generated (lower values indicate better signal quality).

All in all, despite the overlap in the anesthetic agents used, it seems possible to divide the sedated patients to two major groups – patients whose sedation was more propofol-based (PR group) and patients whose sedation was more midazolam based (MD group). Notably (see Table 1) the propofol dose in the PR group was more than 7-fold than in the MD group, and the midazolam dose in the MD group was more than 2-fold than in the PR group. However, this division into major drug groups was used only for a secondary analysis below.

### Control groups

1. Awake control participants were sampled for approximately 10 minutes (with eyes closed in supine position). See sample durations in Table 1 for details on sample durations in the various study groups. They were asked to report thereafter whether they fell asleep and all reported negatively in this regard. 2. Control GA group – patients undergoing abdominal surgery under general anesthesia. These patients received balanced anesthesia. Propofol (1–2 mg/kg), Fentanyl (1–2 mcg/kg) and Rocuronium (0.6–1 mg/kg) were used for induction. After intubation, anesthesia was maintained with Sevoflurane (1 MAC). EEG and BIS were recorded after patient’s intubation until the end of the surgery.

### EEG data analysis

The auditory oddball protocol was employed to evoke brain response. However, the sampled activity was not analyzed in a manner, which was time locked to the stimulus (event related analysis). Rather the continuous EEG during the task was analyzed without reference to stimulus times (task related analysis)^32,33,41,42^.

The raw EEG from 2 anterior electrodes (F3, F4) and 2 posterior electrodes (O1, O2) was filtered to the alpha band-pass (7–13 Hz) (Fig. 1). The 4 samples were divided into 10 second segments and each segment was further divided into 1 second epochs. The sample from each of the electrodes was then evaluated for noisiness at the 1 second epoch level, by computing the standard deviation / mean ratio. Epochs, which involved a ratio greater than 1, were considered noisy^32,33^ and were excluded from further analysis. An epoch was excluded for all 4 electrodes, if it was considered noisy in even one of them. 10-epoch segments, in which more than half (5) of the epochs were excluded as noisy were also excluded from further analysis. Poor EEG signal was defined as samples which included intervals of more than 15 minutes without a valid signal. Samples with poor EEG signal were disqualified from further analysis.

To avoid laterality effects, we then selected, based on overall epoch power, for each valid epoch, the strongest anterior activity, between the F3 and F4 electrodes, and the strongest posterior activity, between the O1 and O2 electrodes. The use of the anterior and posterior stronger activities was meant to overcome potential misplacement of this simple headset and especially rotational misplacement, in which, for example, the left anterior electrode and the right posterior electrodes would be more central (or vice-versa), and therefore may record stronger activity. We then computed the epoch posterior/anterior ratio between these strongest posterior activity and strongest anterior activity. For each segment, we counted, the number of valid (non-noisy) epochs in which the posterior/anterior ratio was greater than 1 – this is the number of valid epochs in which the alpha posterior activity was greater than the alpha anterior activity. We divided this greater posterior activity count by the total number of valid epochs per segment to generate the segment posteriorization/anteriorization index (P/A index). The use of count, instead of average between epochs, was another step to reduce possible noise effect. The P/A index was the median of all valid segment posteriorization indices – using median and not mean was yet again another step in reducing a possible effect of aberrant noisy activity. It should be stressed that all the analysis presented above was automatic without manual intervention. Altogether, the analysis was based the evaluation of whether activity was greater anteriorly or posteriorly in hundreds of 1-second epochs, with all the noise-reduction steps presented above.

### BIS EEG values

BIS values were recorded every 5 minutes by looking at the monitor and immediately documenting the first observed value. The five minutes interval approximates, in terms of analyzed value counts, the number of values sampled by the P/A index after excluding the significant portion of noisy data points, which in the various experimental conditions led to a sample points every 1.5 minutes to every 5 minutes on average (see Table 1 – inter-A/P index interval). It was further intended to mimic practical clinical utilization, in which the anesthesiologist does not attend to the monitor at the sub-minute scale, but rather once every few minutes. The median value from each participant’s samples was used as the BIS value for this participant. The use of a median statistic over the entire procedure is certainly coarse, and furthermore, as the sample is only once in a few minutes, it may lead to lack of sensitivity for rapid fluctuations in both the P/A index and the BIS. Still, the use of a median value, over the entire procedure, gives a sense of the overall level of sedation or anesthesia, which is less sensitive to temporary extreme values. If association can be found for such an overall index of sedation and the occurrence of post-procedural recall, it could be hoped that this association could be finetuned even further in larger patient samples, with evaluation of finer resolution dynamics of the index. Nevertheless, it should be noted that when such an association is not found at the overall level, it might still be found with such finer resolution analysis, which may require a larger sample size.

### EMG/EOG analysis

We measured the level of forehead and ocular muscle activity, and especially of blinking, from the AF3 electrode. Previous studies showed that eye blinks are evident in the low frequency delta range^43^. Therefore, for every epoch, which was validated as described above, we computed the AF3 delta (1–4 Hz) average absolute amplitude. Manual inspection revealed that when the average absolute amplitude is below 10 microvolts it is difficult to differentiate the signal from EEG activity, and when the average amplitude is above 30 microvolts it is difficult to differentiate the signal from noise. Therefore, we included in the analysis only epochs with average activity of 10–30 microvolts. Data from participants with average amplitude outside this range was excluded. For each of the valid samples we then averaged the amplitude of all valid epochs (10–30 microvolts range) to generate the global EMG/EOG index.

### Statistical analysis

The study presented here is a pilot study and we could only relate upon a limited literature regarding the required sample size. Nevertheless, based on some preliminary literature, up to 30% recall under sedation was expected^36^. Hence the study group of the sedation patients were calculated to contain 30 patients with expected 10 recall patients. Evaluation of the sample size of patients undergoing sedation was done according to the formula in appendix 2. This yielded a needed sample of 21 patients under sedation, and for safety we sampled 26 patients. This sample size was matched by the total sample size of the other control groups^44^.

It should be emphasized that the data, which were gathered in this study, were used to calibrate the algorithm for P/A index computation. But, once the index computation was finalized and implemented in software (Matlab), the analysis of all data was re-run automatically. Then, the results from all study participants were collected in Excel, without any manual intervention, and the statistical analysis was determined by a statistician at The Dallah Lana School of Public Health.

The comparisons of both P/A index and BIS, among the multiple study arms, were done with Kruskal-Wallis One Way Analysis of Variance on Ranks (with Dunn’s Method post Hoc analysis for multiple pairwise comparisons). The comparison in the sedated group, between recall and no recall was done using Mann-Whitney Rank Sum Test. The evaluation of the relation between the global EMG/EOG, on the one hand, and P/A index and BIS, on the other hand, was done with linear regression and with Pearson correlation (independent variables = EMG/EOG, dependent variables = BIS or P/A index).

## Results

**Figure 2:**
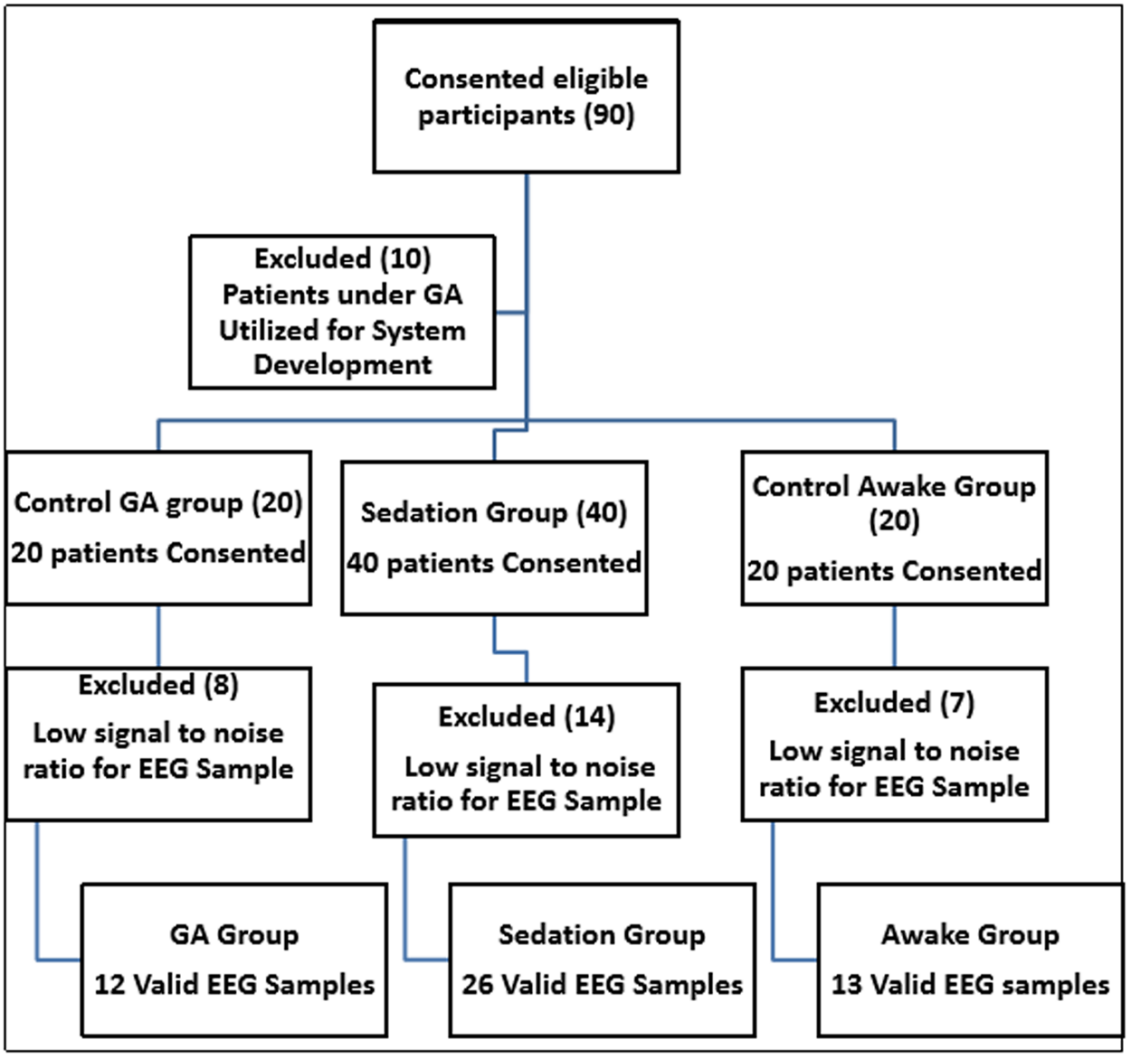
STARD participant flow diagram.

Between January 2017 to June 2018, a total of 90 participants were recruited for this study. Out of them, the 10 first pre-specified participants under general anesthesia were used to refine the experimental set-up and data collection process and thus were not used for the final analysis. Specifically, we had to learn how to use this off-the-shelf headset in the recumbent condition in stable manner and for this aim introduced the use of supportive folded linen under the neck. Forty participants underwent sedation for several procedures (in the cardiac electrophysiology unit – cardiac electrical studies, in the angiography unit – liver chemoembolization and biliary duct drainage), another 20 participants underwent abdominal surgery under GA, and 20 participants were awake volunteers. Poor EEG signals were recorded in 28 participants, who were thus not included in the final analysis (Valid samples; GA group 12/20 patients, awake group 13/20 volunteers, sedation group 26/40 patients) (Fig. 2, STRAD diagram). This poor signal was noticed in patients with thick hair. In addition, manipulations during the surgery, such as Trendelenburg, caused slight movement of the EEG helmet, and resulted in a noisy signal.

The demographics and characteristics of drugs used for sedation in 26 patients are outlined in table #1. Midazolam was the primary drug used for sedation in 18 procedures (median dose/median duration of Midazolam 3 mg/75 min, median dose/duration of Propofol 30 mg/75 min). Propofol was the main drug used for sedation in the other 8 procedures (median dose/median duration of Propofol 235 mg/75 min, median dose/duration Midazolam 1 mg/75 min) (Table #1). Overall, 12 patients out of 26 patients tested positive for recall with the Brice questionnaire. Approximately 2/3 of patients recalled voices and 1/3 recalled pain. All patients who underwent GA tested negative for recall (n = 13). All participants had EEG and BIS recordings.

### Analysis of the P/A index in sedated Patients

**Figure 3:**
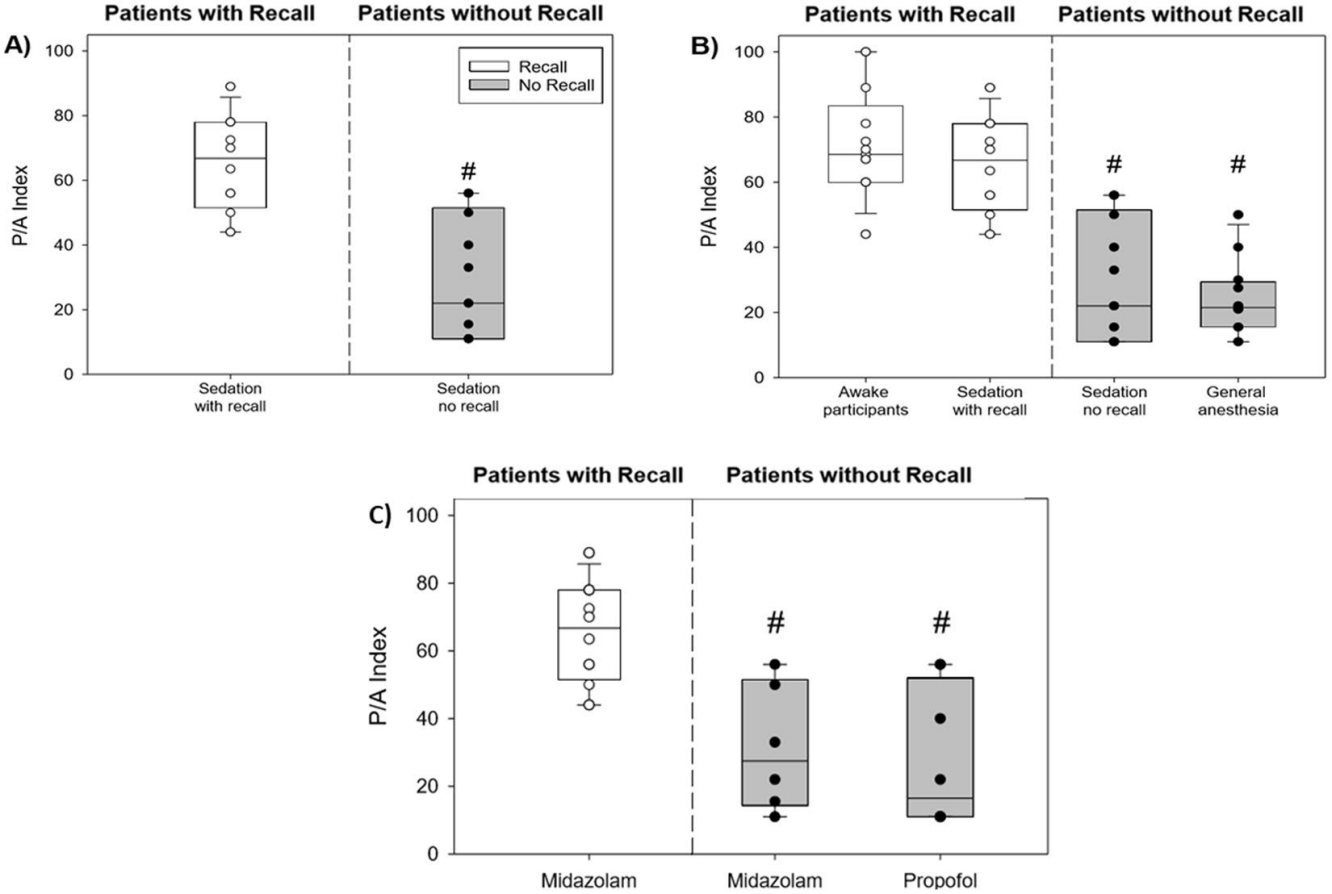
Comparison of P/A Index between study groups. (**A**) Comparison of P/A index in sedated patients with and without recall. For each group the median and interquartile range (IQR) are presented (# - significant difference – see text). (**B**) Comparison of P/A index between all study groups (awake, sedated with recall, sedated with no recall, GA group). (**C**) Comparison of P/A index in sedated patients with and without recall separated according to main anesthetic medication (Midazolam and Propofol).

The P/A index differed under sedation between patients who had recall (median 66.75, interquartile range (IQR) 51.5–78) and patients who did not have recall (median 22, IQR 11–55.5) (Fig. 3a). The difference was evaluated by Mann-Whitney Rank Sum Test and was found significant, p < 0.001.

As an additional evaluation of this new index, and due to the wish to rely on an additional comparison beyond the Brice questionnaire, we also analyzed the index in two additional control groups – one, of awake participants, expected to have recall (and indeed recall was reported by all of its participants) and the other, of patients undergoing general anesthesia, expected not to have recall (and indeed no recall was reported by all of its participants). Figure 3b shows the P/A indices in all the experimental conditions and the overall differentiation between both recall conditions (awake and sedation with recall) on the one hand, and all no-recall conditions (sedation and GA). The differentiation was evaluated by Kruskal-Wallis One Way Analysis of Variance on Ranks and these recall vs no-recall differences were all found significant after Dunn’s Method correction for multiple comparisons (all p’s < 0.01). However, no difference was found between the two recall groups (p≈1) and between the two no-recall groups (p≈1).

Finally, analysis of the effect of the main drug, which was used for sedation, did not show differentiation between midazolam and propofol in the patients who did not show recall, but both groups differed from the sedated patients who had recall (for all of whom the main drug of sedation was midazolam) (Fig. 3c). The statistical significance of this comparison was evaluated by Kruskal-Wallis One Way Analysis of Variance on Ranks and was found significant, p≈0.001. After Dunn’s Method correction for multiple comparisons, the difference between the midazolam no-recall [MD − R] group and the midazolam recall [MD + R} group was significant, p < 0.05. The difference between the propofol [PR] no-recall group and the [MD + R] group was significant, p < 0.05, but there was no significant difference between the [MD − R] group and the [PR] no-recall group (p ≈ 1).

### Analysis of BIS in sedated Patients

**Figure 4:**
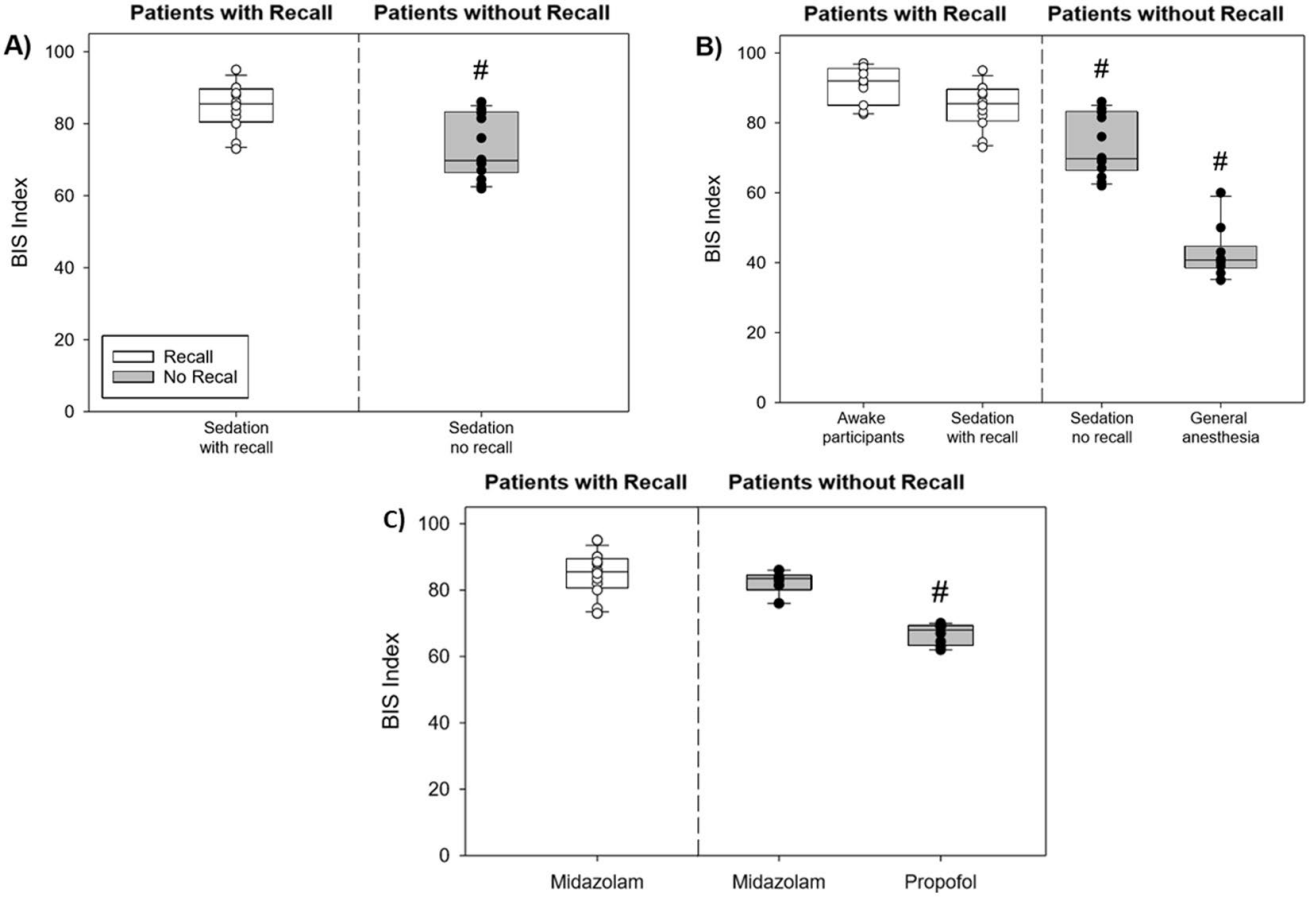
Comparison of BIS between study groups. (**A**) Comparison of BIS in sedated patients with and without recall. For each group the median and IQR are presented (# - significant difference – see text). (**B**) Comparison of P/A index between all study groups (awake, sedated with recall, sedated with no recall, GA group). (**C**) Comparison of BIS in sedated patients with and without recall separated according to main anesthetic medication (Midazolam and Propofol). The difference in BIS between MD + recall to MD no recall is not statistically significance.

For the sake of evaluation of the possible novelty of the above findings with regard to the P/A index, we compared them to a similar analysis of the BIS samples, using Mann-Whitney Rank Sum Test analysis. BIS also differentiated between sedated patients, who had recall, and sedated patients, who did not have recall (Fig. 4a, p≈0.003). Comparing between the various study groups, using Kruskal-Wallis One Way Analysis of Variance on Ranks with Dunn’s Method correction for multiple comparisons, was also significant (Fig. 4b, p < 0.001). However, the difference between the sedation with recall group and the sedation with no-recall group was not significant after the correction (p≈0.27). Still, the differences between the awake patients and both no-recall groups were significant (p < 0.01) and also the differences between the two recall group and the general anesthesia group (p < 0.01).

Finally, when analyzing for the effect of the main drug, using Kruskal-Wallis One Way Analysis of Variance on Ranks with Dunn’s Method correction, we found that only the patients, who were sedated mainly by propofol (and had no recall) differed significantly from the patients who had recall (all sedated mainly with midazolam) (p < 0.001 after correction). The patients with recall under midazolam [MD + R] did not differ significantly from the patients without recall under midazolam [MD-R] (p≈1 after correction for multiple comparisons) (Fig. 4c). However, this analysis should be considered prudently due to the small sample size involved.

### The association with EMG/EOG

Thus, it may be that the P/A index is less related to the drug type in comparison to BIS – at-least when comparing between the impact of midazolam and of propofol. Furthermore, it seems less discriminating than BIS even between awake participants and patients with recall under midazolam, on the one hand, and sedated patients without recall and patients, undergoing general anesthesia, on the other hand. Thus, BIS seems to have a greater discriminatory power between various states, but this greater discrimination may be less related to recall, at-least as far as these initial results are considered. One of the possible contributors to discrimination among the study target and control groups might be muscle activity, which is normal in awake participants, lacking in general anesthesia and might be intermediate in various types of sedation (due to various degrees of reduced movement).

**Figure 5:**
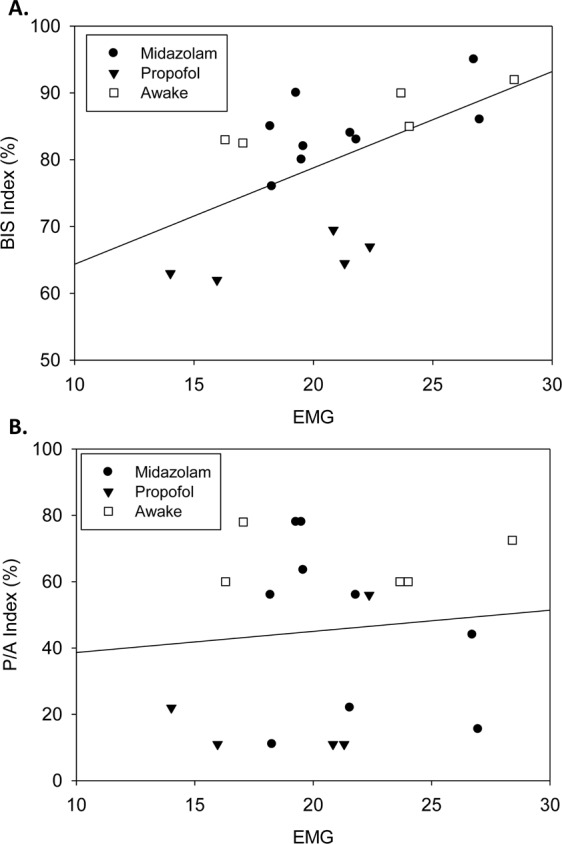
Analysis of the Relationship Between BIS or P/A index and Muscle Activity. Patients under general anesthesia, who were paralyzed, were excluded from this analysis. Utilizing linear regression analysis, a relationship was observed between BIS and EMG activity (Upper R^2^ = 0.311, p = 0.013). The correlation equation is; [y = 1.4x + 49.9], whereas Y equals BIS index and X equals EMG. The P/A index did not correlate with muscle activity (bottom panel, R^2^ = 0.009, p = 0.693). This suggests that muscle activity does not interfere with the EEG signal associated with exteriorization of alpha waves during sedation and general anesthesia.

Thus, to evaluate the effect of the muscle activity on either BIS values or P/A index values we correlated the EMG/EOG with these values (Fig. 5). The GA group was excluded due to low EMG/EOG activity, because of the use of muscle relaxants. An overall correlation between EMG/EOG and BIS was observed (Fig. 5A, R^2^ = 0.311, p = 0.013). By contrast, no correlation was observed between the P/A index and EMG/EOG (Fig. 5B, R^2^ = 0.009, p = 0.693).

## Discussion

This study was a preliminary evaluation of the association of a new evoked index of EEG activity under sedation, based on evoked alpha anteriorization (the P/A index). The index tended to be higher in patients sedated with recall in comparison to patients without recall. A limited secondary analysis suggested that the index may not sensitive to the type of main drug used for sedation (midazolam vs. propofol). Furthermore, it seemed that the P/A index was high in another group of participants, who had recall (awake), and low in another group of participants, who did not have recall (undergoing general anesthesia). Thus, altogether, the P/A index may be associated with recall and possibly less (or not) associated with the specific anesthetic drugs, which were used in the various study groups.

BIS, on the other hand, was shown to offer greater separation among study groups, which may relate to greater richness in monitoring levels of anesthesia. However, BIS did not differentiate somewhat less effectively between recall and no-recall, and especially did not show differentiation between the midazolam recall and no-recall groups. However, the very small sample size should be emphasized in this comparison. Notably, our analysis suggested that at-least some of the fine differentiation among levels of anesthesia by BIS might be related to muscle activity, and thereby less relevant to recall of awareness under sedation. This accords with previous associations of BIS level and muscle activity (EMG) also under general anesthesia^8^.

In our analysis we identified EP patterns in the EEG signal in relation to the auditory Oddball protocol. In previous works we showed that the anterior brain activity is often related to attention processes which may associate with the P3 ERP waves, while the posterior brain activity is more related to earlier perception processes^23,24^. In the ERP literature, light anesthesia may lead to a P3 amplitude decrease and deeper anesthesia to further P3 decrease, but also to the earlier N1 decrease^26^. Hence, light anesthesia might be manifested as a decrease in attention related waves and deeper anesthesia may also involve reduction of perception related waves. The phenomenon of anteriorization of alpha waves under anesthesia might stem from both anterior fragmentation of attention-related activity and deeper anesthesia may also involve posterior decrease of perception-related activity^25–28^. The P/A index stems from this theory. When anesthesia is deeper, the attention related waves fragmentation to faster alpha waves as well as the posterior reduction of perception related alpha activity, leads to a decrease in the ratio between posterior alpha activity to anterior alpha activity^32,45–47^, and the P/A index decreases.

However, while alpha anteriorization is evident with various anesthetic drugs, it is less evident with other drugs^30,48,49^. Therefore, the generality of the P/A index, even still at a feasibility level, would have to be tested with these other drugs as well.

While there are differences in the anesthetic drugs, the use of muscle relaxants and more, there could be hope that an index, which shows the ability to predict recall of awareness in sedation, might be interesting to explore also under GA, due to the lack of effect of muscle relaxants upon it. However, due to the low prevalence of recall under GA, the sample size should be much larger. Even though, there is a growing use of the isolated forearm technique (IFT) to assess the accuracy and the predictability ability of BIS or other indices^7^, the sedated patients might provide another *in-vivo* platform to validate or invalidate indices and to explore the possibly misleading effect of muscle relaxation.

According to the traditional definition, light sedation differs from deep sedation by the ability of the patient to maintain his spontaneous ventilation or the stability of the cardiovascular system^50^. However, as anesthesiologists, it would be of value to be able to differentiate between light and deep sedation according to the effect of anesthetic medications on the brain, and its pharmacodynamic impact on recall of events. A possible characterization of light versus deep sedation, could be defined as the state of recall of awareness. Since our novel index is not dependent on muscle activity, it might be used to identify recall under sedation. Large randomized control trials are needed to validate the reliability of this novel P/A index.

## Limitations

It should be emphasized, first and foremost, that the poor signal-to-noise ratio (SNR) in the current study, using the easy to apply, Emotiv Epoc EEG system prevents any suggestion of real applicability of the study findings at the current stage. First of all, these findings have to repeat themselves with an EEG system, which enables a drastically better SNR, but still is easy to use (low SNR and ease of use is a challenging demand for an EEG system, which involves also posterior electrodes, placed in hair covered areas). Furthermore, the current study is a small pilot proof of concept study. We are currently undertaking additional peer reviewed funded study utilizing a more advanced EEG system, with a better reliability to measure EEG signal, to further assess the validity of our index in sedated patients. Crucially, we would also need to evaluate the impact of other anesthetics, which do not show alpha anteriorization^30^, upon the P/A index, still at the level of a new pilot study at this stage. If, indeed, it would be possible to overcome these two obstacles, of better SNR and of generalization to other drugs, it would be advisable to consider larger scale, blinded RCT studies regarding the efficacy of the P/A index. It should be emphasized that the small sample sizes of the current study, especially for the various sedation groups and their suggested division according to the major drug used, should be considered as merely a preliminary result at the current stage. Other limitations of the current pilot study involve: (i) an uneven division between males and females, with preference to males in all study groups; (ii) younger age of control participants and (iii) shorter sampling duration of awake control patients (but not fewer sampling points, due to better sampling quality – see Table 1).

## Conclusion

We have provided data to support the hypothesis that a novel evoked EEG index can differentiate between sedated patients with, or without recall. Future studies are required, **as stated**, to validate this index and to assess its value as a real-time clinical monitor.

## Supplementary information

Related Manuscript File
