# Supplementary material for: A New Index of Coordinated Posterior and Anterior Evoked EEG to Detect Recall Under Sedation – A Pilot Study
Source: Sci Rep. 2019 Nov 28;9:17859. doi: 10.1038/s41598-019-54270-3 (PMC6883081; doi:10.1038/s41598-019-54270-3)
Supplement: Supplementary file 1 — Related Manuscript File [file 41598_2019_54270_MOESM1_ESM.pdf]

A New Index of Coordinated Posterior and Anterior Evoked EEG to Detect Recall Under Sedation – A Pilot Study

Dana Baron Shahaf MD PhD[1,2], Gregory M.T. Hare MD PhD[2,3,4,5], Andrew J. Baker MD[2,3,4], Violina Chenosia MD[1], Leonid Priven MD[1], Nikhil Mistry, MSc[2], Goded Shahaf MD PhD[6]

1) Department of Anesthesia, Rambam Health Care Campus, Haifa, Israel

2) Department of Anesthesia, St. Michael's Hospital, University of Toronto, 30 Bond Street, Toronto, Ontario M5B 1W8, Canada.

3) St. Michael's Hospital Center of Excellence for Patient Blood Management.

4) Department of Physiology, University of Toronto, Toronto, Ontario, M5S 1A8, Canada.

5) Keenan Research Centre for Biomedical Research, in the Li Ka Shing Knowledge Institute, 209 Victoria Street, Toronto, Ontario, M5B 1T8, Canada.

6) NeuroIndex LTD. Beit Tavor, Yokneam, Israel.

Date: ___________________ Patient Sedation/ GA: ______

Appendix 1: Postoperative Brice Questionnaire

Taken from Chen et al. Amnesia of the operating room in the B-Unaware and BAG-RECALL clinical trials. Anesthesia and Analgesia. 2016.

1. What is the last thing you remember before going to sleep?

- Being in the pre-op area
- Seeing the operating room
- Being with family
- Hearing voices
- Feeling mask on face
- Smell of gas
- Burning or stinging in the IV line
- Other [Free Text]: ____

2. What is the first thing you remember after waking up?

- Hearing voices
- Feeling breathing tube
- Feeling mask on face
- Feeling pain
- Seeing the operating room
- Being in the recovery room
- Being with family
- Being in ICU
- Nothing
- Other [Free Text]: ____

3.Do you remember anything between going to sleep and waking up?

- **-No**
- **-Yes**:-
    - Hearing voices
    - Hearing events of the surgery
    - Unable to move or breathe
    - Anxiety/stress
    - Feeling pain
    - Sensation of breathing tube
    - Feeling surgery without pain
    - Other [Free Text]: ____

Date: ___________________ Patient Sedation/ GA: ______

4.) Did you dream during your procedure?

- No
- Yes
- What about [Free Text]: ______________________________

5.) Were your dreams disturbing to you?

- No
- Yes

6.) Did you experience any nausea or vomiting following your operation?

- No
- Yes

If yes, how many times? __________

7.) What was the worst thing about your operation?

- Anxiety
- Pain
- Recovery process
- Functional limitations
- Awareness
- Other [Free Text]: ____

8.) Are you left or right handed?

- left
- right

9.) Are you a natural red head?

- Yes
- No

10.) Regarding your regular sleep, how often do you remember your dreams?

- Never
- Every few weeks
- At least once a week
- Daily

Date: ____________________ Patient Sedation/ GA: _______

11.) Do you make any effort to remember your dreams?

- No
- Yes

If yes, how?____

Appendix 2: Evaluation of the sample size

Evaluation of the sample size of patients undergoing sedation was done according to the formula presented here, where we assumed the following: 1) The ratio of recall to no-recall would be about 1:2. 2) Sensitivity and specificity would be 80%. 3) Alpha-error was taken to be 0.05. 4) Power was taken to be 80%. This yielded a needed sample of 21 patients under sedation, and for safety we sampled 26 patients. This sample size was matched by the total sample size of the other control groups (45).

$$N_1 = \left\{ z_{1-\alpha/2} * \sqrt{\bar{p} * \bar{q} * (1 + \frac{1}{k})} + z_{1-\beta} * \sqrt{p_1 * q_1 + (\frac{p_2 * q_2}{k})} \right\}^2 / \Delta^2$$

$$q_1 = 1 - p_1$$

$$q_2 = 1 - p_2$$

$$\bar{p} = \frac{p_1 + kp_2}{1 + K}$$

$$\bar{q} = 1 - \bar{p}$$

$$N_1 = \left\{ 1.96 * \sqrt{0.4 * 0.6 * (1 + \frac{1}{2})} + 0.84 * \sqrt{0.8 * 0.2 + (\frac{0.2 * 0.8}{2})} \right\}^2 / 0.6^2$$

$$N_1 = 7$$

$$N_2 = K * N_1 = 14$$
